# The specificity of psychotherapeutic competence: A study protocol on the development of facilitative interpersonal skills across professional pathways

**DOI:** 10.1371/journal.pone.0349702

**Published:** 2026-07-10

**Authors:** Niccolò Fiorentino Polipo, Constanze Maria Springinsfeld, Nina Pfatrisch, Amelie Bihl, Gabor Aranyi, Andrea Jesser

**Affiliations:** 1 Sigmund Freud Private University Vienna, Faculty of Psychotherapy Science, Institute for Training Research, Vienna, Austria; 2 Department of Psychology, University of Copenhagen, Copenhagen, Denmark; School of Nursing Sao Joao de Deus, Evora University, PORTUGAL

## Abstract

**Introduction:**

Facilitative interpersonal skills (FIS) are widely used as an operationalization of therapists’ interpersonal competence. However, the degree to which FIS are specific outcomes of formal psychotherapy training or instead reflect more general abilities developed across different professional pathways or outside of any formal training is unclear.

**Methods and analysis:**

To test whether psychotherapy students show a stronger stage-related increase in FIS levels than students from other fields, this study protocol outlines a cross-sectional cohort design in which FIS levels are assessed at three training stages (entry university level, end of bachelor, end of master) across four professional pathways. The group comparisons include (1) psychotherapy students as the reference group, (2) nursing and (3) teacher education students as comparison groups with high interpersonal demands but without mental health training, and (4) students from the disciplines of Science, Technology, Engineering, and Mathematics (STEM) as a baseline group with low interpersonal demands and no mental health training. We will use linear mixed-effects models with professional pathway and training stage as fixed effects. The primary test will evaluate the fixed effects of the factors professional pathway and training stage, and their interaction.

**Ethics and dissemination:**

Compared to previous studies, our non-clinical, non-interventionist design eliminates any risk of harm to patients and removes variance associated with patient and dyadic factors. Approval has been obtained from the Ethics Commission at Sigmund Freud Private University Vienna. Data and analysis scripts will be shared via open-access repositories. We intend to disseminate results through at least three peer-reviewed publications: primary analyses, secondary analyses, and a theoretical synthesis on implications for psychotherapy training and interprofessional competence.

## Introduction

Proponents of the common factors paradigm have long held that the effectiveness of psychotherapy can, in the main, be explained by factors that underlie most or all therapeutic modalities [1]. This position has been supported by meta-analytic findings showing that, while the average effect of psychotherapy is large and significant, the differences between modalities are small and insignificant [2,3]. Moreover, certain common factors, such as empathy or alliance quality, have been systematically shown to be predictors of treatment success across modalities [4].

Building on this body of evidence, Wampold [5] has proposed the *contextual model*, which views psychotherapy as a social healing practice working through three pathways: (i) the provision of an empathic and emotionally-attuned human relationship; (ii) the creation of positive expectations based on the communication of a plausible rationale for the patient’s distress; and (iii) the promotion of adaptive or healthy actions. The contextual model contrasts with the medical model, which views psychotherapy as a technical intervention defined by modality-specific procedures, rather than general mechanisms of social influence [6].

### From common factors to common skills

If psychotherapy works through general pathways of social influence, as opposed to technical procedures, we can expect therapists to vary in their personal ability to form human connections, or to raise hope in other people. That is why the contextual model predicts that “there will be differences among therapists *within* a treatment,” meaning that some will deliver even the same treatment “more skillfully” than others [55, p. 274, emphasis in original]. This prediction has been supported by research on therapist effects showing that some therapists consistently achieve better outcomes than others [7].

Such findings have sparked an interest in what characteristics make therapists effective [8]. Consistent with the contextual model, research in this area has found that effectiveness is not related to therapist characteristics such as theoretical orientation, professional background (e.g., psychologist, psychiatrist), or even years of experience; instead, it appears to be related to a therapist’s human qualities, such as their interpersonal skills or capacity to question themselves [7].

The current focus on therapist characteristics comes with a reconceptualization of common factors into common skills. For instance, Anderson and colleagues [9] suggest that, rather than referring to “empathy” as one of the “top process-outcome effects” in therapy, we may refer to “the therapist’s *skillfulness* in being empathic” as “*one* of the *indicants* of positive therapist effects” (p. 2, emphasis in original). Similarly, if providing a plausible rationale is one of the factors underlying therapeutic effectiveness [1,10], then this factor can be reframed as the therapist’s skillfulness in being persuasive.

If common factors include therapist factors (e.g., warmth), patient factors (e.g., positive expectations), as well as dyadic factors (e.g., alliance quality; [11]), focusing on skills implies looking at the therapeutic encounter through the prism of the therapist. Instead of being on the patient’s positive expectations, the focus is on the therapist’s capacity to raise them across patients; instead of being on alliance as a dyadic factor, it is on the therapist’s capacity to form alliances across patients. This reconceptualization has crucial implications for training. If, for instance, the working alliance is viewed not only as a treatment factor but also as a *trainable skill* [12], then efforts can be directed towards developing it in trainees.

### Facilitative interpersonal skills

It is in the context of this change of focus from common factors to common skills that Anderson and colleagues [13] have introduced the model of Facilitative Interpersonal Skills (FIS). FIS are a set of eight relational abilities that are considered core competences of effective therapists: (1) *verbal fluency*; (2) *capacity to raise hope or positive expectations*; (3) *persuasiveness*; (4) *emotional expression*; (5) *warmth, acceptance, or understanding*; (6) *empathy*; (7) *alliance bond capacity*; and (8) *alliance rupture-repair responsiveness*.

Although the FIS model is a relatively new operationalization of therapist skills, it synthesizes the long-standing research tradition on common factors, now reframed as common skills. In particular, the eight FIS domains subsume a series of common factors that have garnered solid empirical support for their contribution to outcome, such as empathy, therapist-offered alliance, positive regard, or warmth [14]; as well as a number of common skills grounded in clinical theory and research, such as the therapist’s ability to express themselves verbally, to repair ruptures, to enhance hope, or to persuade others (see [9]).

As a particular set of therapist skills, FIS do not map the whole of psychotherapeutic competence. Other skills have been described in the literature, such as multicultural competences or case conceptualization skills [15,16]. Yet, FIS are the therapist skills for which most evidence exists in terms of their relation to outcome [7,8]. Supporting the meta-analytic findings of the common factors paradigm, FIS have been shown to predict treatment outcomes and positive working alliance [13,17,18].

### The specificity of psychotherapeutic competence

The FIS model, as an operationalization of therapist skills emerging from the common factors paradigm, is rapidly gaining consensus, as “interest in the FIS appears to grow among researchers in different countries” ([19], p. 224; see also [20]). However, an ambiguity seems to lie at the heart of the FIS literature, regarding the conceptual understanding of these skills. *What are FIS*?

On the one hand, FIS are commonly conceptualized as a specific set of professional skills. As argued by Hatcher [21], current models of psychotherapeutic competence “feature relational skills prominently throughout”: interpersonal “expertise” is considered “a goal and an expectation” for the therapist, and its possession a benchmark to safeguard access to the profession (p. 748). In this sense, interpersonal skills are seen not only as a central dimension of psychotherapeutic competence, but even as “the bedrock of professional practice” in this field (p. 748). This position seems substantiated by the association that has been found between FIS and therapeutic outcome. FIS would appear to describe something specific about what makes therapists good at their job.

Yet, on the other hand, skills such as empathy or warmth can hardly be considered the prerogative of therapists, since virtually any helping professional is expected to develop them as well. This is why, contrary to the idea of a specificity of psychotherapeutic competence, it has also been suggested that FIS may be used “by various helpers in role relationships such as medical occupations (e.g., doctors, nurses, technicians), religious/spiritual roles (e.g., priests, bishops, rabbis, shamans), and numerous other helping roles found throughout societies (e.g., hairdressers, bartenders)” ([22], p. 212). This would mean that FIS are *common* not only in the sense that they are “important facilitators of change … across various theoretical orientations” (p. 212), but also in the sense that they are shared beyond psychotherapy, by other professionals.

Although FIS are presumed to be shared across professions, no research has been conducted to test this assumption. In particular, no research has yet explored the degree to which other professionals may perform similarly to therapists in the FIS task. Although hairdressers, for instance, have long been identified as an informal helping group that shows interpersonal skills overlapping those of professional therapists [23], at the same time it may be hypothesized that trained therapists possess those skills to a substantially higher degree. To test the assumption of a specificity of psychotherapeutic competence, then, it would be important to compare the FIS levels of trained therapists with those of other professionals with high interpersonal demands.

However, merely comparing FIS levels across professional categories would make it impossible to disentangle the effects of training from those of pre-existing interpersonal dispositions. The literature on FIS suggests that capacities such as empathy or warmth are not only “professionally cultivated” skills, but also stable, *trait-like* dispositions subject to individual differences ([8], p. 13). It has been theorized that these dispositions may be innate or develop early in life, as a result of contact with attachment figures [21, 24]. Whatever their origin, these dispositions will become entangled with training effects, since they are likely to influence self-selection into professional pathways, with individuals with higher FIS levels, for instance, deciding to pursue a career as a therapist.

Instead, testing the specificity of psychotherapeutic competence means assessing the impact of therapy training on FIS levels *over and above* differences in baseline due to self-selection biases: Accounting for such baseline differences, does formal therapy training develop FIS more than other professional pathways?

### Previous research

What we call the “specificity hypothesis” (H1) refers to the assumption that there is a specific dimension of psychotherapeutic competence, namely an interpersonal or relational one, which formal therapy training would *especially* develop, compared to non-psychotherapeutic training pathways. But what evidence exists from previous research with regards to H1?

In order to corroborate H1, the first point needing to be established is that formal therapy training systematically develops FIS. However, this point is already uncertain. Although it has been shown that FIS can be developed through targeted educational interventions [25–27], studies exploring FIS development as an outcome of standard therapy curricula have led to mixed results. In a cross-sectional study, Salim and colleagues [28] found an association between FIS levels and progression through a five-year university program in clinical psychology (see also [29]). Yet, a longitudinal study by Hill and colleagues [30] found no such association. More studies of this kind are underway [31]. However, the link between therapy training and FIS remains unsure.

The second point needing to be established to corroborate H1 is that therapy training develops FIS *more than* other professional pathways. On this point, the literature is also meagre, and the existing comparative studies mostly rely on outcome measures rather than on direct assessment of FIS.

A series of studies have compared professional therapists to individuals in relatively adjacent mental health roles who have not completed formal therapy training, known as “paraprofessionals” [32,33]. These studies have reported minimal or no differences in treatment outcomes between the two professional groups. As Lambert [34] notes, the fact that “few studies can be found that show the expected superiority” for formally trained therapists calls into question “the value of clinical training and the uniqueness [i.e., *specificity*] of psychotherapeutic interventions taught in graduate schools” (p. 11).

However, to test whether interpersonal expertise is especially developed through therapy training, therapists should not be compared to professionals in mental health roles (e.g., social workers) – who may share partially overlapping training, interpersonal dispositions influencing self-selection into the profession, and on-the-job experience. Rather, they should be compared to professionals *with no mental health training*, but with comparably high interpersonal demands. Yet, studies comparing trained therapists to professionals from other fields are extremely rare.

In one of such studies, known as the Vanderbilt I study, Strupp and Hadley [35] compared a group of highly experienced therapists to a group of college professors who had no formal therapy training, but were known for their interpersonal “warmth, trustworthiness, and interest in students” (p. 1126). A sample of college students with general interpersonal difficulties were assigned to be seen twice a week over 3–4 months by the therapists or the professors, and the professors were instructed to help the patients by “whatever verbal techniques they deemed most helpful” (p. 1127). Patients in both groups experienced significant improvement, and to a greater extent than two control groups. Yet, strikingly, *no significant differences were found in treatment outcomes between the patients treated by the professors and those treated by the professional therapists*.

The Vanderbilt I study was replicated by Burlingame and Barlow [36] with a focus on group (as opposed to individual) psychotherapy. Again, no significant differences were found in treatment outcomes between highly experienced therapists and university professors who did not have any mental health training but were “approached by students for personal advice because of their natural-helper qualities” (p. 458).

More recently, Anderson and colleagues [9] conducted another study inspired by the Vanderbilt I study, which brought the FIS model to bear on the comparison between therapists and other professionals. In this study, 56 doctoral students were screened on the basis of their interpersonal skills, assessed via a self-report questionnaire and a performance task used to measure FIS: the FIS task (see *Measures*). The screening was intended to obtain a dichotomous independent variable (IV) with two levels: a low- vs. high-FIS group. Yet, the study also included a comparison between professional pathways insofar as, among the 23 doctoral students who were thus screened, 11 were training in clinical psychology, while 12 belonged to a variety of non-helping disciplines (e.g., biology) and had no clinical training. Patients from a general university sample were assigned to be treated by the students in the high- or low-FIS group for seven weekly sessions, with untrained students being instructed simply to “enter into a helper role” (p. 8). Patients in the active groups significantly improved, to a greater degree than the ones in a waiting list control group. However, *it was FIS levels (high vs. low) and not training status (clinical psychology vs. non-helping disciplines) that significantly predicted treatment outcomes as well as therapeutic alliance.*

### Limitations of previous research

Previous studies comparing therapists to other professionals [9,35,36] suggest that interpersonal competence may not be unique to trained therapists. These findings are even more striking than the ones from the studies on paraprofessionals, since they provide evidence that not only untrained college professors, but also doctoral students in non-clinical, non-helping careers may perform as well as trained therapists – a result that seems to further dissociate therapeutic skills from training pathways.

However, besides methodological limitations (e.g., insufficient power to detect small-to-medium effects), these studies do not offer a real test of H1. This is because, just like the studies on paraprofessionals, they focused on *treatment outcomes*, not *competence levels*. In the Vanderbilt I study, skill levels were not directly measured. Strupp and Hadley [35] assumed that the professors, peer-nominated for their warmth, would display equal levels of interpersonal skills as the therapists; and the fact that the former turned out to be as effective as the latter was interpreted as confirmation of this. Yet, this assumption remained untested, and Strupp [37] later regretted not having equated the groups on interpersonal skills “*in advance”* (p. 20, emphasis in original).

Skills were not measured in the Vanderbilt I because this study fits within the debate on the nature of therapeutic effectiveness, which dominated psychotherapy research for decades and focused on *treatment factors*, not *therapist skills* [6,7]. Strupp and Hadley [35] wanted to compare common versus specific factors. Their decision to recruit college professors and professional therapists was a means to the end of testing whether the combination of specific *as well as* common factors (assumed to be contributed by the therapists) would yield better outcomes than common factors alone (contributed by the professors). Based on their results, the authors concluded that therapeutic effects observed were driven by the “nonspecific factors inherent in any benign human relationship” (p. 1135).

In contrast, the study by Anderson and colleagues [9] exemplifies the change of focus we have documented from common factors to common skills, insofar as FIS levels were directly measured. Yet, this study still remains somewhat suspended between outcome and competence, since skills were assessed to create a dichotomous IV (high- vs. low-FIS), but the dependent variable (DV) remained the therapeutic outcome. As a result, the study cannot tell us whether significant differences in FIS levels exist between doctoral students in clinical psychology and those in non-helping disciplines, because participants were selected *on the basis of* FIS levels (both trained and untrained students were placed into both the high- and low-FIS groups). While no differences in FIS were reported between trained and untrained students, this is because the selection procedure was designed to control for them, and not necessarily because they are absent in the populations.

Furthermore, no conclusions can be drawn about the effect of training on FIS development from the study by Anderson and colleagues [9] because FIS levels were not assessed *before and after* completion of the respective training programs. As noted, a mere comparison across professional pathways that does not account for baseline differences makes it impossible to disentangle the effects of training from those of “pre-existing interpersonal aptitude and skill” (p. 16) – besides the fact that, in this study, the students in clinical psychology had completed only two years of their doctoral program, so that even the “trained” therapists were not fully trained yet.

### Aims of the study

In contrast to previous studies, our study aims to be the first systematic test of H1 by *using FIS levels as DV and professional pathways (therapy training vs. other professional pathways) as IV, and by assessing FIS levels before, during, and after training.* This change in design represents a conceptual and methodological innovation that is consistent with the shift of focus in contemporary psychotherapy research from treatment factors to therapist skills and allows us to address a different research question than previous studies. Our question is not about “the nature of the psychotherapeutic influence” ([37], p. 18) – i.e., what ingredients drive effective therapy? – but about the “the nature of the therapist’s expertise” (p. 25), i.e., what constitutes the specific competence of professional therapists?

In order to test whether formal therapy training develops the interpersonal skills that are widely considered essential to the professional practice of psychotherapy more than other professional pathways, we will compare four groups: (1) psychotherapy, (2) nursing, (3) teacher education, and (4) Science, Technology, Engineering, and Mathematics (STEM) students. FIS levels will be assessed at three training stages, using independent cohorts of students: at entry to university (T0), at the end of the bachelor (T1), and at the end of the master (T2). This *cross-sectional* design is a pragmatic alternative to a longitudinal study, which would be unfeasibly long (see *Limitations*).

It will allow us to test H1, namely whether FIS levels increase to a significantly higher degree among therapy students than among the other groups, while accounting for baseline differences due to self-selection. Statistically, this corresponds to a significant interaction between professional pathway (therapy, nursing, teaching, STEM) and training stage (T0, T1, T2). Although Anderson and colleagues [9] remain “optimistic” that future studies controlling for pre-training FIS levels will lead to the identification of significant training effects (p. 16), at the same time it is not possible, on the basis of the available evidence, to make any prediction as to whether H1 will be corroborated or falsified. As previously noted, the evidence for the systematic effect of standard therapy curricula on FIS development is mixed, and that on the superiority of therapy training over other professional pathways is entirely lacking.

Besides testing H1, we are interested in tackling secondary research questions such as: (i) whether some training pathways significantly develop certain FIS subdimensions (as opposed to the overall FIS score); (ii) whether different legs of the training contribute to FIS development (e.g., the added value of the master compared to the bachelor); (iii) whether significant differences exist in baseline FIS levels across training pathways, reflecting self-selection effects; (iv) what specific curricular components (e.g., clinical internships, supervision) are associated with FIS development; and (v) whether there is evidence for general trainability of FIS across training pathways (especially nursing and teaching), even if therapists were to show the most pronounced growth.

In summary, the study examines whether FIS differ across professional pathways and stages of training, and whether patterns of change across stages vary between pathways. In particular, the study tests whether stage-related increases in FIS are more pronounced in psychotherapy students than in students from other professional pathways.

## Materials and methods

**Fig 1:**
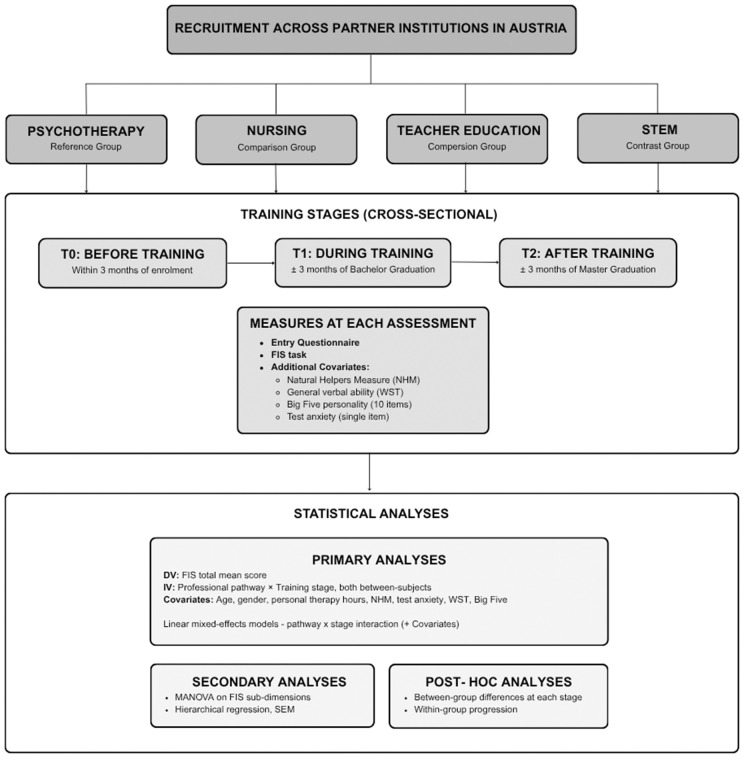
Overview of the study design.

As noted, we will use a comparative, cross-sectional design in which FIS levels are assessed across four professional pathways before, during, and after training. For an overview of the study design see Fig 1. Students at T0 will be assessed within three months of their enrolment in their university program. Students at T1 and T2 will be assessed within three months before or after they graduate from their bachelor and master, respectively, and prior to beginning any form of post-graduate employment. In this way, we aim to separate the effects of training from those of post-graduate, on-the-job professional experience. This is another point on which our study improves on previous research. In the Vanderbilt I study, for instance, therapists differed from professors in that they had not only “*formal training in psychotherapy*,” but also years of “*clinical experience*” ([37], p. 19, emphasis in original). Yet, since the patients consisted of college students, then the professors too had extensive experience directly relevant to the therapeutic task, because of their “day-to-day interaction with many college students over a good many years” (p. 19). For this reason, Hill and Knox [38] note that findings from the Vanderbilt I study highlight the importance of disentangling “the effects of experiential learning versus structured training,” since “people can learn interpersonal skills through informal observation, practice, reflection, and feedback” (p. 799). To separate experiential learning from formal training, we will not only assess FIS levels before the students have acquired significant practice, but also record the extent to which they have engaged in relevant activities alongside their studies (see *Measures*). However, any professional experience that the students may have gained in the context of curricular internships will be considered an integral part of their training program.

Besides professional experience, we will consider the potential effect of personal experiences on FIS development. An example is experiences of mental health problems, both one’s own and those of close others. The literature on the wounded healer model indicates that the development of helping skills, such as empathy, is consequential to overcoming personal struggles [39]. This phenomenon is not unique to therapists but transfers to other professionals, such as nurses with lived experience of the health problems they attend to [40]. Another and related experience is personal therapy. It is commonly held that the experience of being a patient is an important way in which trainee therapists develop their clinical skills [41]. Yet, non-therapists may also have been patients in therapy for many years and may have come out of this experience having developed a sense of how to “talk like a therapist” or relate to someone in suffering. Previous research has neglected the role of personal experiences as possible sources of FIS development. Only Burlingame and Barlow [36] controlled for this variable by selecting professors who had “never sought therapy themselves” (p. 458). Yet, in contemporary Western society, where personal therapy has become increasingly normalized and people are exposed to therapy language through social media [42], it might be unfeasible to screen out participants on the basis of this criterion. A better alternative is to record the extent to which participants are familiar with it and use it as a covariate (see *Measures*).

### Group comparisons

As noted, we are going to compare therapy students to trainees in “disciplines other than the mental health professions” ([36], p. 458). In contrast to Strupp and Hadley [35], who considered only one category of non-therapists (educators), we will use a variety of group comparisons. Yet, contrary to their recruitment strategy, we do not wish to select the representatives of each professional category who display the highest level of FIS, nor do we want to create groups of high- or low-FIS students [9]. Rather, we will recruit the average representatives of each group, so as to be able to tackle the question of whether average differences in FIS development exist between therapy students and trainees in other fields. To limit our scope, we will focus on the impact of *formal academic (university-based) training* on FIS development. Going back to Allen and colleagues (2024), this means that we are going to exclude service professionals with limited or non-academic training (e.g., hairdressers), as well as ministers undergoing faith-based training (e.g., priests). Rather, we will consider only academic programs that are comparable in length (5 years), as they conform to the 2^nd^ cycle of the Bologna system: bachelor (180 ECTS) + master (120 ECTS).

Our groups include a reference group of psychotherapy students, (2) a comparison group of nursing students, and (3) one of teacher education students, both of which have high interpersonal demands but no mental health training, and a baseline group of STEM students, with low interpersonal demands and no mental health training. A detailed description of each comparison group and its rationale follows below.

1. *Psychotherapists* (reference group: high interpersonal demands, formal therapy training): Previous research on FIS development has not always differentiated between students in clinical psychology and psychotherapy, both referred to generally as “therapists” (e.g., [28]). However, in Austria, where data will be collected, the two professions have distinct training pathways, with clinical psychology having a greater focus on theoretical knowledge, assessment, and diagnosis, and psychotherapy having a greater focus on practical knowledge and relationship-building. In this study, we will focus on psychotherapy students, since their pathway is deliberately organized to build interpersonal competence across the arc of training; if any training specificity exists, it should be most visible here. In particular, in contrast to other European countries (e.g., Italy or Belgium) where psychotherapy is a post-graduate specialization to be pursued after studies in psychology (or medicine), in Austria students can obtain a bachelor and master in psychotherapy science as an academic discipline in its own right, which allows a direct comparison with other academic programs conforming to the Bologna system. The training program in psychotherapy in Austria involves three components: (i) *Propädeutikum* (introductory courses); (ii) *Fachspezifikum* (specialized training in a particular psychotherapeutic modality with internships, personal therapy, and supervision); (iii) and general academic training (exams, theses).
2. *Nurses* (comparison group: high interpersonal demands, no mental health training): Building on Allen and colleagues [22], we consider nurses a group of health care professionals whose clinical training may contribute to FIS development, despite not focusing explicitly on mental healthcare or psychotherapy. Compared to medical studies, which place greater emphasis on the acquisition of technical or theoretical knowledge, nursing studies present a similarity with therapy training in that students acquire extensive field experience and patient contact throughout their studies in the context of internships and supervision. Furthermore, there is a well-established literature on the impact of nursing studies on the development of interpersonal qualities such as empathy, which emphasizes the importance of the personal as well as professional development of students in a way that closely matches the literature on therapist development (e.g., [43]). To reduce the overlap with our reference group, we will screen out nurses who have significant mental health training or related internship experiences, such as psychiatric nurses.
3. *Teachers* (comparison group: high interpersonal demands, no clinical training): Building on Strupp and Hadley [35] and Burlingame and Barlow [36], we also include educators as a professional group with high interpersonal demands. Previous studies focused on college professors. Yet, since there is no training program to become a college professor, we will focus instead on primary and secondary school teachers. Teacher training cultivates moment-to-moment responsiveness and relationship management in classrooms, yet it does not include clinical or psychotherapeutic content. While the professors in previous studies taught a variety of subjects, we will select students who specialize in humanistic or discursive fields (e.g., history), as opposed to technical or number-based fields (e.g., mathematics), to maximize contrast with the STEM group.
4. *STEM* (contrast group: low interpersonal demands, no clinical training): This group of professionals with low interpersonal demands serves as a baseline for interpreting FIS development in more relationally oriented pathways. It corresponds to the group of doctoral students in non-helping fields in the study by Anderson and colleagues [9], which allowed the authors to “assume” that the students’ “interpersonal skills were not necessarily the result of training experiences” (p. 3). However, while Anderson and colleagues used a heterogeneous group of students from various fields, including humanistic and discursive fields (which may bear on some FIS subdimensions), we will create a more homogeneous group of students in technical and number-based fields that are not expected to be associated with FIS development, such as physics and technical chemistry.

### Statistical analyses

To test our primary hypothesis (H1), we will use linear mixed-effects models (LMMs). The main predictors will be *professional pathway* (four levels: psychotherapy, nursing, teacher education, STEM) and *training stage* (three levels: T0, T1, and T2). Since participants are sampled cross-sectionally at each stage, both predictors will be modeled as between-subjects factors (i.e., with no repeated measures within individuals). The primary test of H1 will be whether there is a significant *interaction effect* between the two factors. An interaction indicating that FIS levels increase to a significantly higher degree across training stages in psychotherapy students than in the other groups would provide evidence in support of the specificity hypothesis. Since we have several study programs within some training pathways (e.g., technical chemistry and physics in the STEM group), programs may differ in baseline FIS or in how they train interpersonal skills. To account for this, models will include a random intercept for study program (nested within pathway).

All models will include theoretically relevant covariates that may be associated with FIS development, such as hours of personal therapy, natural helping ability score (see *Measures*), age, and gender (see *Gender-related aspects*). Age is an important covariate because training pathways may have different lengths. Although the official length of therapy training is five years, for instance, the average length of completion is closer to eight years. Besides reflecting a difference in the quantity of training received, variable program length can introduce maturational factors (e.g., a 28-years-old having acquired more life experience, and possibly emotional maturity, than a 25-years-old). Excluding students who have had gap years may reduce generalizability and make recruitment harder. Instead, age will be included as a covariate in all analyses to account for potential differences in general developmental and life-experience-related factors across groups. On the other hand, we will exclude second-career students (e.g., therapy trainees with a background in social work or education as first profession). Besides age, additional covariates (e.g., test anxiety; see *Measures*) will be included.

If the interaction effect is significant, we will conduct post-hoc tests to examine between-group differences at each stage (e.g., pre-training differences across pathways at T0) and within-group progressions (e.g., added value of the master compared to the bachelor among therapy students). Furthermore, while H1 focuses on the *overall* FIS score, secondary analyses will be conducted to test whether professional pathways significantly differ in the development of specific FIS skills. To this purpose, we will run a multivariate analysis of variance (MANOVA) using the eight FIS subdimensions as DVs and correcting for multiple testing. As secondary analyses, we will also explore specific predictors of FIS development (e.g., curricular components such as internships or clinical supervision) using hierarchical regression and/or LMMs in order to better understand the specific mechanisms that facilitate FIS acquisition. Finally, we will use structural equation modeling (SEM) to model FIS as a latent construct, thereby accounting for measurement error.

### Participants and recruitment

Power was estimated using a two-way fixed-effects ANOVA model in G*Power as an approximation to our planned LMMs. Assuming α = 0.05, power = 0.80, and a medium effect size (Cohen’s *f* = 0.25), the required total sample is 279 participants, corresponding to ca. 23 participants per cell in the 4 (professional pathways) x 3 (training stages) design. To enable more demanding secondary analyses (e.g., MANOVA, regressions, SEM), we plan to recruit ca. 30 participants per cell (360 total). If resources permit, we will increase to 36 per cell (432 total), which would provide power to detect small-to-medium effects (*f* = 0.20).

Students will be recruited through partner institutions in Austria. Psychotherapy students will be recruited at the Sigmund Freud University (SFU) Vienna through the Bachelor and Master in Psychotherapy Science. Nursing students will be recruited at UMIT TIROL – Private University for Health Sciences and Health Technology through the Bachelor’s degree in Nursing Science (in cooperation with the Health University of Applied Sciences Tyrol) and the Master’s degree in Advanced Nursing Practice. Students in primary and secondary teacher education studies will be recruited at the University College of Teacher Education in Lower Austria (PH NÖ), the Private University of Education, Diocese Linz, and the University of Education Carinthia. Finally, students in STEM fields will be recruited at the University of Vienna (physics) and the Technical University Vienna (technical chemistry).

The invitation to take part in the study will be circulated through program mailing lists and in-class announcements by contact people in each partner institution. Participants will not receive course credits for their participation. However, to ensure feasible recruitment across all groups each participant will receive a giftcard for a large retailer (e.g., MediaMarkt) upon study completion. The study will be presented as an opportunity to learn about one’s interpersonal skills while contributing to a scientific project. Participants will also be offered a personalized feedback report interpreting their FIS scores. Previous studies suggest that feedback may work as another factor of motivation. In the study by Salim and colleagues [28], for instance, almost all students (97%) requested it. Questionnaires will be administered using SoSci Survey [44] and the FIS task will be administered via an online platform (see *Measures*). Each participant will complete measures once. Codes will be used to identify participants while preserving anonymity.

### Measures

*Entry questionnaire*. Basic sociodemographic information, such as age and gender, will be recorded through a self-report questionnaire. Additionally, the entry questionnaire will record the student’s training pathway (psychotherapy, nursing, teacher education, STEM), study program (e.g., physics, technical chemistry), training stage (entry-level, bachelor, master), academic performance, and general satisfaction with studies (for bachelor and master graduates only). For relevant pathways (e.g., psychotherapy, nursing, teacher education), we will also focus on specific curricular components, such as hours spent in practical internships and direct or indirect patient or student contact. In the case of therapy students, we will also record the psychotherapeutic modality they have chosen as part of their *Fachspezifikum,* as well as the number of hours spent in “self-experience” activities (i.e., personal therapy in the context of training) and clinical supervision, their kind of patient contact (e.g., co-therapy, shadowing), and their kind of internship experiences (e.g., psychiatric ward, rehabilitation clinic, private outpatient center). Finally, the entry questionnaire will ask students about their personal and professional experience outside of their academic studies. As concerns the former, the questionnaire will ask about experiences of mental health problems and adverse life events (first-hand, in a family member, or in a significant other) and experience with personal therapy unrelated to therapy training (e.g., therapeutic modality, number of hours). As concerns professional experience, we will ask students to what degree they have been involved in activities that may be directly relevant to the FIS task, such as student jobs, hobbies, or volunteering activities in role relationships (e.g., mental health helplines, church-related activities).

*FIS task.* Our DV will be assessed using the German version of the FIS task developed by Berning and colleagues (2024) [45]. The FIS task is a validated performance test involving standardized video stimuli depicting challenging therapeutic interactions, to which participants respond as if they were the therapist [46,47]. The video stimuli were originally created on the basis of the data collected from one of the Vanderbilt studies (Vanderbilt II), by identifying challenging moments in therapy which were then re-enacted by actors [9,13]. Since trainees are unreliable in assessing their own interpersonal skills, the main advantage of a performance task is that it reduces bias in self-assessment [48]. Eight short videoclips are presented successively in a random order, and participants verbally respond to each clip. The responses are video-recorded and filters are used to make the participants’ faces unrecognizable, ensuring blinded rating. Although researchers are exploring the use of machine learning to code the FIS responses, human rating remains more reliable [49]. Each study participant’s recordings will therefore be evaluated by two trained, independent raters using the FIS task coding manual (5-point scale from low level (1) to high level (5) of interpersonal skills) assessing each of the eight FIS subdimensions [13]. Raters will be blind to the participants’ professional pathway or training stage. The FIS total mean score will be used for analyses.

*Natural helping ability*. The *Natural Helper Measure* (NHM) by Stahl and Hill [50] will be used to measure the students’ inclination for interpersonal relationships, referred to as “natural helping ability.” This self-report questionnaire includes the following 5 items, rated on a 7-point Likert scale from never (1) to always (7) and averaged into a total score: “I often find myself helping others with their problems;” “I have been told that I am good at helping others;” “I have been told that I would be a good counselor/therapist;” “I consider myself to be ‘naturally’ good at helping others;” “I am comfortable helping others with their problems.” Despite obvious limitations with the self-report assessment of helping ability, the NHM score will be included as a covariate to account for dispositional factors independent of training. Furthermore, we will administer a single item devised by Stahl and Hill [50] to assess motivation to help others: “I plan to pursue a career in a helping profession,” rated on a 7-point scale from very unlikely (1) to very likely (7).

*Additional covariates*. To account for other individual differences that may influence FIS performance, we will include three additional covariates. In line with Salim and colleagues [28], we will assess test anxiety using a single-item measure (“During exams, I feel a great deal of tension”; [51]), rated on a 5-point scale from strongly disagree (1) to strongly agree (5). We will also include a measure of general verbal ability, using the *Wortschatztest* (WST; [52]). The WST is a standardized multiple-choice vocabulary test widely used as a measure of crystallized verbal intelligence in German-speaking populations. It consists of 42 items, where participants are presented with one real word and five non-words and must identify the real word. Finally, we will include a 10-item scale of personality based on the Big Five model to account for traits such as Neuroticism and Extraversion [53].

### Status and timeline

Ethics approval has been obtained in October 2025 and is valid for three years after notification (Ethics Commission of the Sigmund Freud Private University; ID: BDMFE7EACLED6D91890). Participant recruitment has not yet started, no data have been collected, and no results have been generated. Recruitment and data collection are expected to start in September 2026 (testing of entry-level students at the start of the winter semester 2026) and will proceed on a rolling basis across sites and training stages until the prespecified target sample size is reached. We expect recruitment and data collection to be completed by October 2027 at the latest. FIS responses will be independently rated by two trained raters shortly after task administration. A complete, scored dataset suitable for quantitative analysis should be available in February 2028. Immediately after, we will conduct the primary and secondary analyses (see *Statistical analyses*), with initial results expected in summer 2028. In the final phase of the project, starting in July 2028, we will focus on disseminating the results (see *Dissemination*).

### Ethical aspects

Approval has been obtained from the Ethics Commission at Sigmund Freud Private University (ID: BDMFE7EACLED6D91890). The outlined study is in accordance with the European Code of Conduct for Research Integrity. All data will be anonymized and stored securely. Individual results will remain confidential and not be shared with the students’ partner institutions. All participants will sign informed consent forms and will have the right to withdraw at any point without penalty.

In previous studies involving professionals from non-clinical fields [9,35,36], patients were assigned to be treated by untrained “therapists.” As acknowledged by the authors, this poses obvious ethical problems. Our study avoids this by using FIS levels, not patient outcomes, as DV. Our non-clinical, non-interventionist design not only eliminates any risk of harm to patients, but also removes variance associated with patient and dyadic factors.

The main ethical considerations in this study instead pertain to the student participants. In addition to potential temporary test anxiety elicited by the FIS task, some participants may react negatively to a low score through feedback, especially if the skills assessed are central to their personal or professional identity. To mitigate this risk, participants will be informed that the FIS task is a context-dependent research instrument and that the results do not represent a definitive assessment of their abilities or professional potential. Feedback on performance will be provided only upon request and in a standardized, non-evaluative format, in order to minimize the risk of misinterpretation or undue self-criticism. Furthermore, test anxiety will be assessed using a single-item measure; participants indicating elevated levels (i.e., “agree” or “strongly agree”) will be provided with general information about available student support services.

### Gender-related aspects

The gender distribution across the chosen professional pathways is uneven: Students in psychotherapy and nursing are predominantly female, whereas students in STEM fields are predominantly male. Such imbalances may influence the results, as there are theoretical and empirical reasons to expect gender-related variation in interpersonal skills. Socialization processes, gender norms, and biological factors may shape some of the FIS dimensions, such as emotional expression and empathy. Research has shown that women, on average, tend to score higher on some measures of these dimensions [54,55]. However, while some measures of interpersonal skills show differences, evidence for gender-based differences on the FIS task specifically is limited. A conference poster [56] hints at differences in empathy, but its details are unpublished. Most published studies have not found or reported significant correlations between gender and FIS performance [9,17,57,58].

A solution would be to purposefully sample participants to achieve balanced groups. Yet, artificially matching groups on gender could obscure meaningful group-level differences, compromise the representativeness of our sample, and raise practical and ethical issues in selection. Therefore, we will retain the natural distributions across training pathways to reflect real-world enrollment patterns and preserve ecological validity. We will record gender via the entry questionnaire, transparently report group compositions, use it as a covariate in all models, and consider gender-related variation in the interpretation of our results.

### Data management and data availability

All study data will be collected, stored, processed, and shared in accordance with applicable data protection regulations (GDPR) and institutional policies. The research dataset will not contain directly identifying information (e.g., names, private contact details). Participants will be assigned a pseudonymous study ID, which will be used to link questionnaire data and FIS task responses. If contact details are required for study administration (e.g., compensation), these will be stored separately from the research data, accessible only to authorized personnel, and deleted as soon as they are no longer required. The key linking study IDs to any administrative information will not contain personal identifiers and will be stored separately from the research data.

Questionnaire data will be collected via the secure online platform SoSci Survey [44]. The FIS task will be administered via a purpose-built secure online platform. Participants’ task responses will be video-recorded and stored on secure university servers with access restricted to authorized project personnel. To ensure blinded rating, videos will be processed prior to rater access (e.g., visual filters and pitch shifting).

Data will be retained for at least ten years following publication, in line with institutional policies and good scientific practice. Prior to data sharing, datasets will be anonymized/deidentified to minimize the risk of reidentification.

As this manuscript describes a study protocol, participant recruitment has not yet started, no data have been collected, and no results have been generated. Therefore, no dataset is currently available. Deidentified quantitative data supporting the findings of the future study (e.g., FIS scores and questionnaire data), together with accompanying documentation (codebook and analysis scripts where possible), will be made available via an established open-access repository (e.g., OSF or Zenodo) upon publication of the study results. Due to the highly sensitive nature of video data and the associated ethical and legal restrictions, raw video recordings cannot be made publicly available. Public data deposition would breach the conditions of the ethics approval and participant consent. Access to such data may be considered upon reasonable request, subject to ethical approval, legal constraints, and a data use agreement.

## Discussion

As the first study comparing the effect on FIS development of therapy training vs. other professional pathways, our project tackles a fundamental question on the nature of psychotherapeutic competence that has not been addressed before. Is interpersonal expertise a central dimension of psychotherapeutic competence, one that specifically requires formal therapy training to develop? Or, said otherwise, just how “common” are the common skills that have been identified through decades of research on what makes therapists effective? The conceptual and methodological innovation that allows our study to tackle this question is making competence levels the central focus (DV), as opposed to treatment outcomes.

Ironically, the question investigated by our study is the one that was raised by the Vanderbilt I study, but which this study could not answer. On the one hand, in fact, the results from the Vanderbilt I study – and of the subsequent ones conducted in its wake – are said to have “yielded major implications for our thinking about” formal therapy training, since they appear to “call into question the very necessity” of such training ([38], p. 798–799). Indeed, if individuals with high levels of interpersonal skills but “no psychotherapy training” can be “as effective in helping clients” as professional therapists ([59], p. 328), then what does this say about the value of therapy training? And yet, on the other hand, the Vanderbilt I study was actually “not directly investigating the effects of training” (p. 798). The “equivalent results” of the Vanderbilt I study have “garnered considerable attention to implications of training,” but at the same time its design “specifically was *not* a test of the practical effects of training” ([9], p. 3, emphasis in original), as recognized by Strupp [37] himself. Similarly, the study by Anderson was not “intended to provide a general assessment of training” (p. 16).

Our study will be the first to provide evidence that is directly relevant to H1. If our results corroborate H1, they will provide long-awaited empirical evidence for the specificity of psychotherapeutic competence. But if they falsify H1, they will not only raise the need to fundamentally rethink the nature of psychotherapeutic competence, but also challenge the rationale at the basis of therapy training. If nursing or teacher education studies can develop FIS to a non-inferior degree than formal therapy training, and if FIS are the skills that drive therapeutic effectiveness, then “what would be the justification for intensive and protracted [therapy] training” ([37], p. 18)? Such a null result might indicate, for instance, that in standard therapy training, interpersonal “qualities have taken a back seat to cognitive abilities and measures of traditional academic performance” ([9], p. 17), which would call for a systematic restructuring of such programs. Or, alternatively, another way to interpret a null result would be to call into question the validity of the FIS model, as an operationalization of therapist skills, or of the FIS task, as a method for assessing them. This outcome would also have far-reaching implications for the field of psychotherapy research since, as noted, consensus is growing around the FIS approach. In all of these different scenarios, the results from our study would contribute empirical findings to important basic and applied debates that go to the very foundations of the field.

### Limitations

The most rigorous way to evaluate the impact of training on FIS development would be an experimental design in which students are randomly assigned to professional pathways, thereby controlling for pre-training differences in interpersonal dispositions, and to follow the development of FIS longitudinally. However, this design is not feasible under the present conditions, not least due to the duration of professional training (5 + years). A cross-sectional cohort design with three training stages (T0, T1, T2) therefore represents a pragmatic alternative. Its main limitation is that it cannot capture potential self-selection processes over time – for example, that students with certain levels of FIS may be more likely to (dis)continue training. To partially address this issue, dropout rates will be recorded and considered in the analyses. Importantly, findings will be interpreted with caution: stage-related differences will not be taken as direct evidence of developmental change, but rather as indicative of patterns consistent with either training-related development or selection effects, which cannot be fully separated within this design.

A second limitation concerns the context specificity of the FIS task, as its video stimuli are situated in a therapeutic setting with which psychotherapy students may be more familiar than students in other pathways. This may advantage psychotherapy students and thus influence group comparisons. In interpreting the results, we will therefore explicitly consider the possibility that observed differences reflect contextual familiarity rather than differences in underlying interpersonal skills. At the same time, several considerations qualify this limitation. First, the FIS task is currently the only validated performance-based measure of facilitative interpersonal skills. Second, since the mobilization of such skills is inherently context-dependent, meaning that there is no way of capturing FIS as a context-free phenomenon, the therapy setting remains the obvious context of choice given the study’s focus. Third, as noted, results from our study could initiate a discussion on the validity and scope of the FIS task which, despite being widely praised, is bound to have limitations. Finally, in view of the normalization of “therapy culture” [42], a familiarity with the therapy setting is not a prerogative of trained therapists anymore, as laypeople are increasingly exposed to it through either their personal therapy or public discourse. Thus, while a contextual bias may exist, it will be taken into account when interpreting group differences, without reducing observed differences to familiarity with the therapeutic context alone. To partially account for differences in contextual familiarity, prior exposure to psychotherapy (e.g., hours of personal therapy) will be included as a covariate to examine the extent to which group differences are attenuated. These analytical and interpretative considerations will inform a cautious and differentiated interpretation of the findings.

### Dissemination

We will disseminate findings through peer-reviewed publications reporting (i) the primary hypothesis test, (ii) secondary analyses (e.g., FIS subdimensions), and (iii) a theoretical synthesis on implications for psychotherapy training and interprofessional competence. Results will be presented at international and national conferences, including the annual meeting of the Society for Psychotherapy Research. Data and analysis scripts will be shared via open-access repositories.
