# Supplementary material for: The specificity of psychotherapeutic competence: A study protocol on the development of facilitative interpersonal skills across professional pathways
Source: PLoS One. 2026 Jul 10;21(7):e0349702. doi: 10.1371/journal.pone.0349702 (PMC13353991; doi:10.1371/journal.pone.0349702)
Supplement: Attachment — Submitted filename: Response to Reviewers.docx [file pone.0349702.s002.docx]

**Reviewer #1: Overall, the protocol addresses an important and timely question concerning the specificity of psychotherapeutic competence and the extent to which Facilitative Interpersonal Skills (FIS) develop through formal training in psychotherapy, as compared with other professional pathways. The research question is clearly articulated, the conceptual framework is well grounded in the literature, and the proposed comparative design has the potential to contribute relevant empirical evidence to an ongoing debate within psychotherapy research. The use of the Facilitative Interpersonal Skills (FIS) task as the principal outcome measure represents a notable strength, as it provides a performance-based assessment of interpersonal competence. Furthermore, the inclusion of multiple comparison groups (psychotherapy, nursing, teacher education, and STEM fields) enhances the interpretability of the findings, and the proposed statistical approach appears, overall, appropriate for testing the stated hypothesis.**

**However, several aspects of the protocol would benefit from further clarification:**

**-Line 36: The phrase should be reformulated, and the abbreviation should be defined at its first occurrence in the abstract. For example: students from the disciplines of Science, Technology, Engineering, and Mathematics (STEM);**

Thank you for this helpful suggestion. We have revised the wording accordingly and now define the abbreviation at its first occurrence in the abstract (lines 37–38).

**-Although the authors appropriately acknowledge limitations related to the cross-sectional nature of the study design and the potential contextual bias arising from the use of FIS stimuli specific to the therapeutic context, it would be helpful to provide greater clarification regarding how these issues will be taken into account in the interpretation of the results.**

Thank you for this helpful comment. We agree that the original version did not sufficiently clarify how these limitations will be taken into account in the interpretation of the results. We have now revised the manuscript accordingly.

Specifically, we clarify how both (a) potential self-selection mechanisms (lines 659–662) and (b) contextual familiarity with the therapeutic setting as a source of bias (lines 666–668 and 698–704) will be considered in interpreting the findings.

**Reviewer #2: An excellent topic, with very pertinent questions. The rationale for the study is very comprehensive; however, given the way the ideas are presented, it would be important to try to summarise the main objectives of the study.**
Thank you for this helpful suggestion. We have added a concise summary of the study’s main objectives at the end of the Introduction, under the section “Aims of the study” (lines 296–299).

**- Study design – too broad, with too many variables. A diagram of the study design should be included to make it clearer for readers.**

Thank you for bringing this issue to our attention. To improve clarity, we have added a schematic diagram of the study design (see Figure 1 cited in lines 302-303). The design follows a cross-sectional cohort approach, combining four professional pathways with three training stages. FIS performance serves as the primary outcome, while sociodemographic and experiential variables (e.g., age, gender, prior therapy exposure) are included as covariates. The main analysis tests pathway × stage effects to examine whether patterns of FIS differ systematically across training contexts.

**- Age is a very important covariate, as the population includes therapists/teachers as well as students, which will introduce a bias into the study.**

Thank you for highlighting the importance of age as a covariate. We would like to clarify that our sample consists exclusively of students across four different training pathways, and does not include fully trained therapists or teachers. However, we agree that age remains a crucial variable in this context.

As outlined in the manuscript (lines 419–425), training pathways differ in their typical duration and structure, and students may enter or complete their studies at different stages of life. As a result, age may capture differences in general developmental and life-experience-related factors that are independent of formal training. To account for this, age will be included as a covariate in all analyses. In addition, we will examine the extent to which group differences are attenuated when adjusting for age, allowing for a more differentiated interpretation of whether observed effects are more likely attributable to training-related versus maturational influences. We have further clarified this point in lines 425–426.

**- Ethical issues – how will participants react to the results, how will their behaviour be modified, and in cases where participants score highly on anxiety, will they be referred to other professionals?**

Thank you for raising these important ethical considerations. We have clarified this aspect in the revised manuscript.

Participants will receive feedback on their FIS performance only upon request and in a standardized, non-evaluative format. It will be explicitly communicated that the FIS task is a context-dependent research instrument and that the results do not represent a definitive assessment of their abilities or professional potential, in order to minimize potential negative effects on self-perception or behaviour (lines 561–563).

We would like to clarify that the study does not include a clinical assessment of anxiety. Test anxiety is assessed using a single-item measure (“During exams, I feel a great deal of tension”), which serves as a contextual covariate rather than a diagnostic tool. Participants indicating elevated levels (i.e., “agree” or “strongly agree”) will be provided with general information about available student support services. This has been clarified in lines 563–565.
